# In-hive variation of the gut microbial composition of honey bee larvae and pupae from the same oviposition time

**DOI:** 10.1186/s12866-019-1490-y

**Published:** 2019-05-24

**Authors:** Zuzana Hroncova, Jiri Killer, Josef Hakl, Dalibor Titera, Jaroslav Havlik

**Affiliations:** 1 Department of Microbiology, Nutrition and Dietetics, Czech University of Life Sciences Prague, Kamycka, 129, 165 00 Prague, Czech Republic; 2 Department of Genetics and Breeding of Farm Animals, Institute of Animal Science, Pratelstvi, 815, 104 00 Prague, Czech Republic; 3 Institute of Animal Physiology and Genetics, v.v.i., Academy of Sciences of the Czech Republic, Videnska, 1083, 142 20 Prague, Czech Republic; 4 Department of Agroecology and Crop Production, Czech University of Life Sciences Prague, Kamycka, 129, 165 00 Prague, Czech Republic; 5 Bee Research Institute, Dol 94, 252 66, Libcice nad Vltavou, Czech Republic; 6 Department of Zoology and Fisheries, Czech University of Life Sciences Prague, Kamycka 129, 165 00 Prague, Czech Republic; 7 Department of Food Science, Czech University of Life Sciences Prague, Kamycka 129, 165 00 Prague, Czech Republic

**Keywords:** Actinobacteria, Bacteroidetes, Black pupae, Firmicutes, Gammaproteobacteria, Honey bee larvae, qRT-PCR

## Abstract

**Background:**

Knowledge of microbiota composition, persistence, and transmission as well as the overall function of the bacterial community is important and may be linked to honey bee health. This study aimed to investigate the inter-individual variation in the gut microbiota in honey bee larvae and pupae.

**Results:**

Individual larvae differed in the composition of major bacterial groups. In the majority of 5th instar bees, Firmicutes showed predominance (70%); however, after larval defecation and during pupation, the abundance decreased to 40%, in favour of Gammaproteobacteria. The 5th instar larvae hosted significantly more (*P* < 0.001) Firmicutes than black pupae. Power calculations revealed that 11 and 18 replicate-individuals, respectively, were required for the detection of significant differences (*P* < 0.05) in the Bacteroidetes and Firmicutes abundance between stages, while higher numbers of replicates were required for Actinobacteria (478 replicates) and Gammaproteobacteria (111 replicates).

**Conclusions:**

Although sample processing and extraction protocols may have had a significant influence, sampling is very important for studying the bee microbiome, and the importance of the number of individuals pooled in samples used for microbiome studies should not be underestimated.

**Electronic supplementary material:**

The online version of this article (10.1186/s12866-019-1490-y) contains supplementary material, which is available to authorized users.

## Background

Bees contribute to agricultural productivity and profitability. In the recent past, the increasing reduction in honey bee populations and beekeepers has been a matter of great concern worldwide, necessitating more research into the maintenance of colony health [1]. Currently, most studies are focused on the microbiota of the digestive tract, which have been proposed to play a role in the honey bee health [2–7]. The bacterial spectrum of a honey bee is affected by its interactions with individuals that comprise a colony, age, diet, and developmental stage. Additionally, the hive and the pollination environment have their own characteristic microbiota differing from that of the bee hindgut [8–11].

Individuals of social insects orally share food (trophallaxis); this is generally perceived as a factor leading to the homogenisation of microbial profiles among individuals within a single colony [9, 12–16]. Minor variations of the bacterial spectrum within the same colony might reflect the health status and short-term differences in the physiology or ontogenetic stage of individual bees [17]. This homogeneity is often taken for granted when designing studies and determining the number of individuals to be sampled. The number of sampled individuals pooled in studies focusing on larval microbiota often varies between 4 and 10 individuals [18–25].

The microbial spectrum changes during ontogenesis and honey bee and bumble bee larvae have been shown to have different bacterial profiles compared to adult bees [26]; this is because, unlike the segmented digestive tract of the adult honey bee gut, the developing larvae only have a midgut, which is connected to the hindgut at the pre-pupal stage [27]. The larval gut presents an ideal environment for pathogens which infect the larval stages of bees [28–31]. The 5th instar larvae host up to eight bacterial clades [32] but there is no clear evidence on the dominant groups and the main drivers underlying microbial balance. Recent research suggests that Firmicutes (Firm) are the most prevalent taxon in this group [32], while another study [33] demonstrated the dominance of Gammaproteobacteria (Gamma). Bacterial counts decrease and low Gamma counts persist in the gut after defecation and during pupation, serving as a proxy for subsequent bacterial colonisation after morphogenesis [34]. Other studies suggest that the gut is devoid of microbiota and that the digestive tract is re-inoculated after this stage [8]. Gaining specific knowledge regarding the dynamics and variation of the larval gut microbiome is of importance for two main reasons: first, it is required for the design of robust study protocols; and second, the larvae are the focus of probiotic applications, aiding in defence against pathogens and influencing colony health [22, 35].

Despite the existence of reports on honey bee gut microbiota, information on the microbial communities present in the digestive tract of larvae and pupae is conflicting and inconsistent. The aim of this study was to investigate the variation in the composition of microbiota in individual larvae and pupae. We used denaturing gradient gel electrophoresis profiling (DGGE) and quantitative real time polymerase chain reaction (qRT-PCR) to compare the gut microbiota of two developmental stages (5th instar and black pupae) of *Apis mellifera carnica* in individuals from one hive. Power analysis was used to demonstrate that pooled samples can reduce the number of analyses required. The honey bee gut microbiota might act as a simplified model for studying the gut microbiota of higher animals, and a uniform and reproducible experimental design would therefore be of use.

## Results

### Inter-individual microbiota variations

**Fig. 1:**
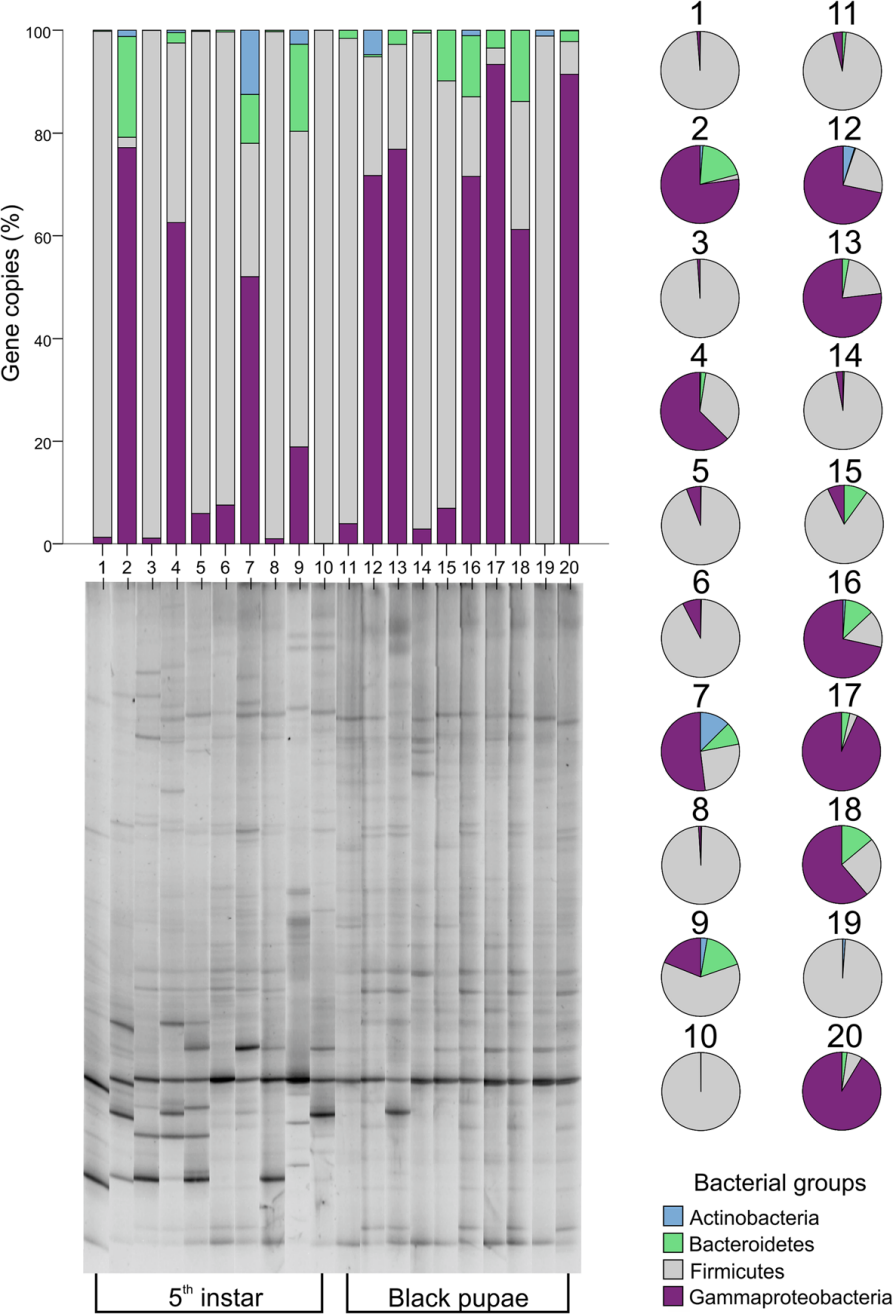
Quantitative real time PCR analysis of the abundance of selected bacterial groups in total gastrointestinal tract samples from individual honey bees at different ontogenetic stages and the corresponding DGGE profiles. The number of copies of the 16S rRNA gene per 1 g of the honey bee (5th instar and black pupae) gastrointestinal tract scaled to 100% is shown on the y-axis

The presence of Gamma, Firm, Act, and Bct bacteria was examined in the undissected guts of individual bees in the morphogenetic stages of 5th larval instar (LF3) and black pupae (PB). To illustrate the microbial variability between individuals, denaturing gradient gel electrophoresis profiling was done. The qPCR results showed that Firm were the predominant group in the majority of individuals in the 5th instar (70%) (Fig. 1), while Gamma bacteria were most abundant in the gut of 30% of larvae. In samples 1, 3, 8, and 10, more than 98% of the bacterial population was from the Firm clade. Act bacteria was observed in the digestive tract of one sample (sample 7). Individuals 2 and 9 hosted 17–20% Bct species (Fig. 1), which was clearly more than in the other samples. After larval defecation and during pupation, Firm counts decreased to 40% of the original bacterial counts, and were outnumbered by Gamma, which corresponded to 60% of the bacterial population. Two black pupae (17 and 20) were predominantly colonised by Gamma (90%), while the Firm clade formed the major proportion in individuals 11, 14, and 19 (94–99%). These differences in the abundance and prevalence of bacterial species are likely due to the highly specialised metabolic niches in the gut, where these species are localised, as is commonly observed in other animal microbiomes [35].

### Effect of morphogenetic stage on microbiota

**Fig. 2:**
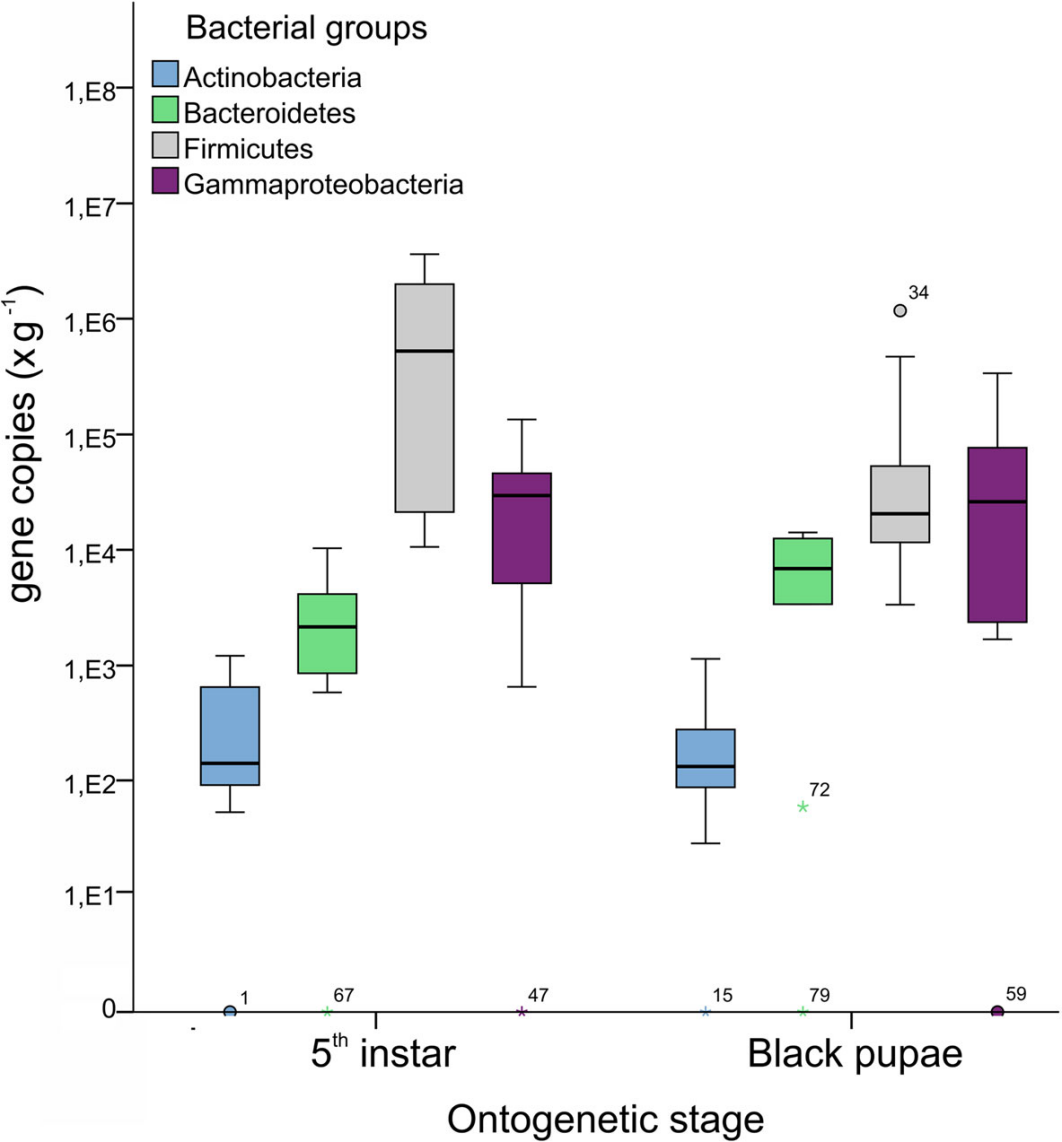
Boxplot of quantitative real time PCR data for the averaged abundance of selected bacterial groups from total gastrointestinal tract samples of honey bees at different ontogenetic stages. Log-transformed numbers of copies of the 16S rRNA gene per 1 g of the honey bee gastrointestinal tract are shown on the y-axis. Boxes depict the average abundance of Actinobacteria, Firmicutes, Gammaproteobacteria, and Bacteroidetes from total gastrointestinal tract samples of 5th instar and black pupae

The current study also revealed the effect of age and morphogenetic stage on the gut microbial composition. Results showed that the 5th instar hosted significantly (*P* < 0.001) more Firmicutes than black pupae. In the digestive tract of 5th instar larvae, Firm bacteria were markedly more numerous than Gamma bacteria, by nearly two orders of magnitude (1.8 × 10^6^ Firm vs. 4.0 × 10^4^ Gamma; the numbers are the means of 16S rRNA gene copies per 1 g of digestive tract content); Bct were more numerous than Act (3.3 × 10^3^ Bct vs. 3.6 × 10^2^ Act) (Fig. 2). In black pupae, the distribution between Firm and Gamma groups was more balanced (1.8 × 10^5^ Firm vs. 9.5 × 10^4^ Gamma) while Bct were more abundant than Act (6.9 × 10^3^ Bct vs. 2.8 × 10^2^ Act); the inter-individual variation was lower than in the guts of the 5th instar larvae.

### Power analysis

Power analysis revealed that the number of replicates required for the detection of significant differences (*P* < 0.05) between the two evaluated developmental stages varied considerably among bacterial groups (Table 1). Variability between stages was much lower for Bct (standard deviation SD LF3/PB = 3369/5067) than for the other groups; a very low number of replicates was therefore required for this group. Standard deviations (LF3/PB) for Firm, Act, and Gamma were 2,811,784/377051, 403/363, 46,485/111054, respectively. A comparable power analysis of data by Hroncova et al. [34] has showed that the analysis of pooled samples could reduce the required replicate number for almost all bacterial groups. The inclusion of inter-colony variability as another tested factor further reduced the required replicate number for Act, from 242 individuals to 66; for Gamma, from 23 to 6 bees; and for Bct, from 405 to 102. More replicates were needed for the two-way ANOVA (24) than for the one-way ANOVA for Firm alone (13).

**Table 1:** Power analysis for determination of the required number of replicates needed to clarify the differences of bacterial groups (Actinobacteria, Firmicutes, Gammaproteobacteria, and Bacteroidetes) between developmental stages (target test power 0.90, α < 0.05)

	One colony, current study (one-way ANOVA)	Four colonies, Hroncova et al. [34] (one-way ANOVA)	Four colonies, Hroncova et al. [34] (two-way ANOVA)
Actinobacteria	478	242	66
Firmicutes	18	13	24
Gammaproteobacteria	111	23	6
Bacteroidetes	11	405	102

## Discussion

A detailed understanding of the overall function and persistence of the bacterial community in honey bees is necessary before probiotics can be introduced in the beekeeping practice. Our results indicate differences in the microbial composition of honey bee larvae and pupae from the same oviposition time. We focused on the 5th instar because developing larvae have a discontinuous gut (i.e., the foregut is not connected to the hindgut) [27]; larvae are targeted by many major pathogens, and infected individuals die at the 4th to the 6th instar [28, 31, 36, 37]. In addition, the microbiota harboured by the 5th larval instar of *A. mellifera* is more diverse than that in later stage insects [26, 34]. The biggest differences in microbiota were between the bacterial groups Firm and Gamma; in the majority of 5th instar bee larvae, Firmicutes were the predominant group (70%); after larval defecation and during pupation, the abundance of Firmicutes decreased to 40%, while that of Gammaproteobacteria increased. These differences may be associated with individual changes in nutrition and metabolism, genetic specificities, and random ecology factors. The presence of the appropriate microbial composition in this instar might be crucial later in the development; a well-established community within the gut may preclude infection by potential pathogens via either space-exclusion or nutrient competition [8]. Such exclusion effects have been previously documented, with studies correlating the presence of bifidobacteria and other lactic acid bacteria strains with the absence of the pathogens *Melissococcus plutonius* and *Paenibacillus larvae*, respectively [5, 38]. Similarly, the presence of the newly described bacterium *Parasaccharibacter apium* increases larval resistance to *Nosema* [39]. As suggested by Cremer et al. [40, 41], foods could be enriched with regard to specific non-pathogenic potentially probiotic microbes, which can inhibit pathogen growth. This is particularly important for honey bees, as they are susceptible to several destructive diseases [42, 43] attacking the brood. However, as bacteria may be supported by faeces deposited on comb surfaces and interaction networks within the hive and colony, considerable variations may exist in microbial communities between individuals [44]. Conversely, the distribution of the roles within the hive, such as foraging for food and nest material, nest defence, or food storing [45, 46], inspired the notion that the honey bees share microbiota and that their microbial diversity is low [17, 26, 47–53]. This might not be the case, however. Interactions among honey bees may lead to an accumulation of bacterial species from hive materials in the blind gut, as well as that of some species usually found in the gut of adult bees; however, both the composition and abundance of the larval gut microbiota seem erratic [8, 26, 32, 34, 54]. Nonetheless, even workers of the same age within a colony can harbour widely differing proportions of the core gut bacterial species [17, 24, 44]. Information with regard to such interactions and inter-and intra-colony microbial variation is lacking, particularly for the pupal stages. In agreement with our previously published data [34], we herein confirmed that total bacterial counts tended to decrease (*P* = 0.098) following larval defecation and during pupation. However, no significant differences were noted at the *P* < 0.05 level; consequently, differences in microbial counts within a single hive could not be compared with those observed in previous studies where bees from multiple hives were examined. Our results revealed that the 5th instar hosted significantly (*P* < 0.001) more Firmicutes than black pupae; thus, the ontogenetic stage of the honey bee could be an important factor affecting changes in gut microbiota. Firm bacteria were also more abundant than Gamma bacteria in the digestive tract of the 5th instar larvae, by nearly two orders of magnitude. It is thus evident that the relative proportions of the core microbiota in the colonies vary with the age of the insects [34] and between years [18]. However, these shifts have been poorly explored in previous studies, partly because it is not possible to compare bacterial profiles reported for different studies used different protocols. The experimental design, particularly with regard to sampling, should be uniform because most studies have relied on pooled samples from several bees, and considerable individual differences exist in the abundances of bacterial groups in the digestive tract of bees. Moreover, the limited experimental design and use of pooled samples is likely to have resulted in strain diversity or rare phylotypes being missed by most studies performed to date. With regard to sample size, the power analysis performed in the current study revealed that the number of required replicates (individual bees in a pooled sample) varied considerably among bacterial groups (Table 1). A comparable power analysis of data by Hroncova et al. [34] clearly demonstrated that the analysis of pooled samples may potentially reduce the required replicate number for almost all bacterial groups. Another factor that reduced the required replicate number for Act, Gamma, and Bct was the inclusion of inter-colony variability as another tested factor; more replicates were needed for the two-way ANOVA than for the one-way ANOVA only for the Firm group. It is also necessary to consider other sources of sample variance, such as genetic, individual, or ecological variability. One may hence conclude that each animal is a unique individual and experimental design is of the utmost importance in the study of the bee microbiome, mainly with regard to sampling, with the use of pooled samples where necessary to reduce the number of analyses required. As demonstrated by our results, this approach not only ensured the use of acceptable *p*-values, but also generated trends or nonsignificant results indicative of the originality of our research. In deed there are alternatives that address study design or sample size much more directly than significance testing does; but none of the statistical tools should be taken as the new magic method giving clear-cut mechanical answers [55].

## Conclusions

Recent studies have indicated that the social behaviour of the honey bee creates consistent associations of bacteria in their digestive tracts. In contrast, our study provides new and interesting insights into the diversity of some microbial groups inhabiting the honey bee gut. As anticipated, we observed high variation in the abundance of bacterial phyla (Gamma, Firm, Act, and Bct) in the guts of two morphogenetic stages of the same age; 5th instar larvae hosted significantly (*P* < 0.001) more Firmicutes than black pupae. In addition, the presence of the same bacterial groups worldwide in the honey bee supports the hypothesis that these bacteria play a central role in honey bee biology. In this regard, the variations observed between main species clusters may influence colony health. In the present study, we focused specifically on sampling owing to previous work demonstrating that sample pooling can effectively help to reduce the required number of replications. The required replicate numbers were calculated as illustrative example for certain studies.

## Methods

### Honey bee samples

*A. mellifera carnica* at two developmental stages (10 5th instar bees and 10 black pupae) were sampled individually from a single hive at the Bee Research Institute at Dol after receiving permission for use of the private land from the owner (Czech Republic; 50°12′ 23.9“ N 14° 21’ 58.8” E). The genetic background of the individuals was not determined; hence, they might have been super sisters (sharing both a queen mother and a drone father), with a coefficient of genetic relationship of 0.75, or half-sisters (sharing only a mother), with a coefficient of genetic relationship of 0.25 [56, 57]. Sampling was conducted on July 31, 2012. Bee management and samples represented traditional beekeeping practices in the Czech Republic. The authors are solely responsible for the employed ethical approach. The field studies did not involve endangered or protected species.

The 5th instar corresponded to 6-day-old larvae, more precisely, to the last feeding stage LF3 prior to sealing, with the gut completely filled with a yellowish material. Black pupae with dark brown eye pigmentation or when they showed medium thorax pigmentation (Pdm) were sampled [58, 59]. The individuals were collected into disposable tubes and immediately frozen on dry ice. The entire tube-like digestive tracts were collected from each larva or pupa and homogenized, and random 50 mg samples of the homogenized gut from each individual were used for isolation of the total bacterial DNA using the ZR Faecal DNA MiniPrep kit (Zymo Research, Irvine, CA, USA) [34].

### Real-time PCR analysis

Bacterial DNA was quantified using the MX3005P thermocycler (Stratagene, La Jolla, CA, USA), based on the 16S rRNA gene copy numbers (Additional file 1). The following specific primers were used: for Gamma, 1080γF and γ1202R; for Bacteroidetes (Bct), 798cfbF and cfb967R; for Firm, 928F-Firm and 1040FirmR; and for Actinobacteria (Act), Act920F3 and Act 1200R [60]. IBM SPSS Statistics ver. 20 (IBM, Armonk, NY, USA) was used for descriptive data analysis and for the visualisation of qRT-PCR data. Differences between developmental stages were determined by one-way ANOVA for total bacterial counts and by repeated measurement ANOVA for bacterial groups. Based on the study results (means of developmental stages and their variation estimates), we performed a power analysis of the single factor design to determine the required number of replicates needed in each developmental stage to achieve the target test power of 0.90 with α < 0.05. To ensure repeatability of the results in other hives, a similar power analysis was also performed using the data of the comparable developmental stages (5th instar corresponded to LF3; black pupae corresponded to Pdm) from experiment 1 (EXP1) published by Hroncova et al. [34] where an identical methodical/sampling approach was used allowing direct comparison. In this analysis, we determined the required number of replicates for pooled samples in four colonies (single- or two-factor analyses). All analyses were carried out using the STATISTICA program (StatSoft, Tulsa, USA) [61].

### Denaturing gradient gel electrophoresis

The total bacterial community DNA was amplified by targeting the 200-bp partial 16S rRNA gene sequences with universal bacterial set of primers 338GC and RP534 [61], which actually amplifies the genetic information of the honey bee microbiota [34]. PCR products were analysed on a DGGE gel (gradient from 35 to 65%) according to the method of Mrazek et al. [62].

Appropriate standards containing a mixture of PCR products of five known microorganisms [34] were loaded in the centre of gels to minimise gel variability using multi-gel comparisons in the BioNumerics 6.6 software (Applied Maths, Sint-Martens-Latem, Belgium) with the settings used by Hroncova et al. [34].

## Additional file

Additional file 1: Quantitative real-time PCR (qRT-PCR) data of the abundance of selected bacterial groups of honey bees (5th instar and black pupae). Data are log-transformed copies of the 16S rRNA gene per 1 g of the total gastrointestinal tract of individual and pooled samples. Individuals were collected at two developmental stages (10 5th instar individuals and 10 black pupae) from a single hive. Pooled samples show data of the comparable developmental stages from 4 locations and 3 hives at each location (EXP1) published by Hroncova et al. [34] where identical methodical/sampling approach was used allowing direct comparison of results. (XLSX 79 kb)

## Abbreviations

Act: Actinobacteria
Bct: Bacteroidetes
DGGE: Denaturing gradient gel electrophoresis
EXP1: Experiment 1
Firm: Firmicutes
Gamma: Gammaproteobacteria
LF3: Last feeding stage
PB: Black pupae
Pdm: medium thorax pigmentation
qRT-PCR: Quantitative real time polymerase chain reaction
SD: Standard deviation
